# Movement disorders with neuronal antibodies: syndromic approach, genetic parallels and pathophysiology

**DOI:** 10.1093/brain/awx189

**Published:** 2017-09-25

**Authors:** Bettina Balint, Angela Vincent, Hans-Michael Meinck, Sarosh R Irani, Kailash P Bhatia

**Affiliations:** 1 Sobell Department of Motor Neuroscience and Movement Disorders UCL Institute of Neurology, Queen Square, London WC1N 3BG, UK; 2 Department of Neurology, University Hospital, Heidelberg, Germany; 3 Neuroimmunology Group, Nuffield Department of Clinical Neurosciences, John Radcliffe Hospital, Oxford, UK

**Keywords:** neuronal antibodies, movement disorders, chorea, parkinsonism, ataxia

## Abstract

Movement disorders are a prominent and common feature in many autoantibody-associated neurological diseases, a group of potentially treatable conditions that can mimic infectious, metabolic or neurodegenerative disease. Certain movement disorders are likely to associate with certain autoantibodies; for example, the characteristic dyskinesias, chorea and dystonia associated with NMDAR antibodies, stiff person spectrum disorders with GAD, glycine receptor, amphiphysin or DPPX antibodies, specific paroxysmal dystonias with LGI1 antibodies, and cerebellar ataxia with various anti-neuronal antibodies. There are also less-recognized movement disorder presentations of antibody-related disease, and a considerable overlap between the clinical phenotypes and the associated antibody spectra. In this review, we first describe the antibodies associated with each syndrome, highlight distinctive clinical or radiological ‘red flags’, and suggest a syndromic approach based on the predominant movement disorder presentation, age, and associated features. We then examine the underlying immunopathophysiology, which may guide treatment decisions in these neuroimmunological disorders, and highlight the exceptional interface between neuronal antibodies and neurodegeneration, such as the tauopathy associated with IgLON5 antibodies. Moreover, we elaborate the emerging pathophysiological parallels between genetic movement disorders and immunological conditions, with proteins being either affected by mutations or targeted by autoantibodies. Hereditary hyperekplexia, for example, is caused by mutations of the alpha subunit of the glycine receptor leading to an infantile-onset disorder with exaggerated startle and stiffness, whereas antibodies targeting glycine receptors can induce acquired hyperekplexia. The spectrum of such immunological and genetic analogies also includes cerebellar ataxias and some encephalopathies. Lastly, we discuss how these pathophysiological considerations could reflect on possible future directions regarding antigen-specific immunotherapies or targeting the pathophysiological cascades downstream of the antibody effects.

## Teaser

Movement disorders are a common feature of autoantibody-associated diseases. Balint *et al.* provide a novel phenotype-oriented algorithmic approach to guide diagnosis. They review the underlying pathophysiology and point out parallels between immune-mediated and genetic conditions presenting with similar phenotypes. They consider future directions such as personalised immunotherapy.

## Introduction

Neuroimmunology is a rapidly evolving field, fuelled by the discovery of new autoantibodies and syndromes (Lancaster and Dalmau, 2012; Irani *et al.*, 2014). Movement disorders are a prominent and common feature in many autoantibody-mediated neurological diseases, with an expanding spectrum of autoantibodies, but there is a need to establish a phenomenological approach to guide categorization and diagnosis in clinical practice. Although these disorders were generally considered rare and precise prevalences are unknown, it has emerged that, for example, NMDAR antibodies are the most frequent single cause of encephalitis under the age of 30 years (Gable *et al.*, 2012).

These disorders can be encountered by general neurologists or movement disorders specialists alike and it is imperative not to miss these potentially treatable disorders, which can also be an alert to an occult neoplasia. An early diagnosis is important for the prognosis, yet many patients are misdiagnosed, or diagnosed late (Irani *et al.*, 2013; Titulaer *et al.*, 2013). With adequate treatment (immunosuppression or immunomodulation, tumour treatment as appropriate), many patients show a good recovery, although lasting deficits may occur (McKeon *et al.*, 2013; Titulaer *et al.*, 2013; Balint *et al.*, 2014*a*). Often, prolonged and aggressive immunotherapies are required, which carry a risk of serious adverse effects (e.g. toxicity, infections) and significant expenditure to health systems. Hence, rapid recognition and improved therapies are urgently required.

In this review, we outline the spectrum of movement disorders related to neuronal autoantibodies, highlight useful pointers to these conditions, and present a syndromic approach to guide antibody testing. We also discuss the underlying pathophysiological mechanisms, the emerging parallels to genetic movement disorders, the interface between neuroimmunology and neurodegeneration, and conclude on future therapeutic perspectives.

## The clinical spectrum of movement disorders and neuronal antibodies

In movement disorders, the recognition of a characteristic clinical presentation, its phenomenological categorization, and syndromic associations guide the diagnostic work-up. In genetic movement disorders, for example, the plethora of new genes has prompted a phenotype-oriented algorithmic approach (Stamelou *et al.*, 2013; Balint and Bhatia, 2015). A syndromic approach in this context has been to define movement disorders as either ‘isolated’, when occurring alone, or as ‘combined’ when there are associated features (Balint and Bhatia, 2015; Edwards *et al.*, 2016). This allows one to narrow down the differential diagnosis of a particular syndrome, and is necessary because one gene can cause different phenotypes and one phenotype may be caused by different genes.

The situation is similar in the growing number of neuronal autoantibody-associated diseases, where movement disorder may occur in isolation, or, more frequently, combined with other signs, ranging from gross encephalopathy with altered consciousness to more subtle findings like a neuropathy.

We propose an approach for immune-mediated disorders related to neuronal, glial, or ganglioside antibodies, based on the main movement disorder presentations and the concept of isolated versus combined presentations. First, we discuss the phenotypes and point out red flags for the differential diagnosis in order to distinguish them from degenerative, genetic or infectious diseases. Table 1 provides a summary and a reference for clinical practice to guide antibody testing: it allows one to select an antibody panel based on a movement disorder phenotype, age of onset, and the presence or absence of other neurological signs. Table 2 lists the antibodies together with their associated clinical spectra, and, where appropriate, tumour association. It also indicates relative frequencies, to allow assessments of relative pretest probabilities. The section ‘Approach to antibody testing’ highlights some considerations related to test methodology.

**Table 1:** From syndrome to serology: different movement disorder presentations with the main associated neuronal, glial and ganglioside antibodies

Antibody target	Onset		Features		Clinical details
	Childhood	Adulthood	Isolated	Combined	
**Chorea and dyskinesia**					
CV2/CRMP5		+		+	Typically combined with cognitive decline, neuropathy, optic neuritis, myelitis; MRI: often FLAIR hyperintensities (white matter, basal ganglia, temporomesial)
Hu		+		+	Typically combined with gastrointestinal pseudoobstruction, sensorineuronal hearing loss; MRI: often FLAIR hyperintensities (white matter, basal ganglia, temporomesial)
CASPR2		+	+	+	Chorea preceding or combined with behavioural changes
LGI1	+	+	+	+	Chorea preceding or combined with cognitive impairment and encephalopathy; typically in (later) adulthood
NMDAR	+	+	+	+	Chorea or characteristic orofacial and limb dyskinesias; truly isolated presentations are rare, mostly combined with ataxia (in children), neuropsychiatric symptoms, epilepsy, or other signs of encephalopathy
Neurexin-3α		+		+	Mild orofacial dyskinesia combined with encephalopathy with epilepsy, altered consciousness, memory deficits, psychomotor agitation
GABA_A_R	+	+	+	+	Chorea as part of an encephalopathic syndrome with epilepsy, behavioural or cognitive problems or reduced consciousness, can be combined with ataxia or dystonia; MRI: frequent T_2_-weighted hyperintensities
D2R	+		+	+	As part of encephalitis in children, or in ‘Sydenham’s chorea’
IgLON5		+		+	Combined with prominent sleep behaviour disorder and bulbar symptoms; possible additional features: cognitive decline, ataxia, dysautonomia, central hypoventilation, oculomotor disturbance
**Dystonia**					
CV2/CRMP5		+		+	Combined with other signs of encephalopathy
Ma2		+		+	Combined with other signs of encephalopathy
NMDAR	+	+	+	+	Combined with other signs of encephalopathy (e.g. behavioural changes, epilepsy); rarely, hemidystonia or dystonia of neck and larynx as the most prominent symptom in children and young adults
GABA_A_R	+	+		+	Dystonia as part of an encephalopathic syndrome with epilepsy, behavioural or cognitive problems or reduced consciousness, can be combined with ataxia or chorea; MRI: frequent T_2_-weighted hyperintensities
D2R	+			+	Combined with other signs of encephalopathy in children
**Myoclonus**					
LGI1		+		+	Combined with other signs of encephalopathy, important mimic of Creutzfeldt-Jakob disease
CASPR2		+			Myoclonus affecting stance and gait, mainly in elderly males, combined with neuropsychiatric, cognitive or neuropathic symptoms
DPPX	+	+		+	Combined in a multifocal encephalopathy, red flag: gastrointestinal symptoms (particularly diarrhoea)
Neurexin-3α		+		+	Combined with other signs of encephalopathy, resembling encephalitis with NMDAR antibodies
Ri		+		+	Adult paraneoplastic OMS^a^
Ma2		+		+	Adult paraneoplastic OMS^a^
Zic4		+		+	Adult paraneoplastic OMS^a^
Hu		+		+	Adult paraneoplastic OMS^a^
Yo		+		+	Adult paraneoplastic OMS^a^
CV2/CRMP5		+		+	Adult paraneoplastic OMS^a^
VGCC		+		+	Adult paraneoplastic OMS^a^
GAD		+		+	OMS^a^ without underlying malignancy
GQ1B		+		+	OMS^a^ without underlying malignancy
NMDAR	+	+		+	OMS^a^ without underlying malignancy
GABA_A_R	+	+		+	OMS^a^ without underlying malignancy
DPPX	+	+		+	OMS^a^ without underlying malignancy
GABA_B_R	+	+		+	Paediatric and adult cases of OMS
GlyR	+	+		+	Myoclonus typically as part of → combined SPSD, rarely in OMS^a^
**Parkinsonism**					
D2R	+			+	Vary rare: combined with other signs of encephalopathy, in children
NMDAR	+	+		+	Combined with other signs of encephalopathy
LGI1		+		+	Combined with other signs of encephalopathy
CRMP5		+		+	Combined with other signs of encephalopathy
Ri		+		+	Combined with other signs of encephalopathy
DPPX		+		+	Combined with other signs of encephalopathy
Ma2		+		+	Subacute parkinsonism / PSP phenotype with supranuclear gaze palsy (vertical > horizontal) and constant eye closure resembling apraxia of lid opening, combined with additional signs of limbic, diencephalic or brainstem encephalitis, myelopathy or radiculoplexopathy; red flags: hypothalamic-pituitary endocrine dysfunction, weight gain, prominent sleep disorders; MRI: T_2_ hyperintensities of pons, midbrain, thalamus, basal ganglia, cerebellar peduncles, hypothalamus, amygdala, or temporal lobe; sometimes only atrophy or no abnormalities
IgLON5		+		+	Combined with prominent sleep behaviour disorder and bulbar symptoms; possible additional features: gait instability and supranuclear gaze palsy (PSP phenotype); other oculomotor disturbance, cognitive decline, dysautonomia, central
**Ataxia**					
GAD	+	+	+	+	Isolated or combined with SPSD, focal epilepsy, limbic encephalitis; often preceding episodes of brainstem or cerebellar dysfunction; often organ-specific autoimmunity (diabetes, thyroiditis, vitiligo, pernicious anaemia)
CASPR2		+	+	+	Isolated ataxia or combined with encephalopathy with seizures and cognitive impairment
DPPX	+	+		+	Combined with encephalopathy; red flag: gastrointestinal symptoms (particularly diarrhoea)
NMDAR	+	+		+	Combined with other signs of encephalopathy; ataxia is more frequent in children
IgLON5		+		+	Combined with prominent sleep behaviour disorder and bulbar symptoms; possible other features: chorea, cognitive decline, dysautonomia, central hypoventilation, oculomotor disturbance
VGCC		+	+	+	Paraneoplastic cerebellar degeneration (mostly lung cancer), isolated or combined with Lambert-Eaton syndrome or limbic encephalitis
Yo/CDR2		+	+	+	Paraneoplastic cerebellar degeneration (gynaecological tumours), isolated or combined e.g. with brainstem encephalitis, neuropathy
Hu/ANNA-1		+	+	+	Paraneoplastic cerebellar degeneration (mostly lung cancer) combined with limbic or brainstem encephalitis, myelitis or neuropathy
Ri/ANNA-2		+	+	+	Paraneoplastic cerebellar degeneration combined with limbic or brainstem encephalitis, myelitis,
PCA2		+	+	+	Paraneoplastic cerebellar degeneration combined with limbic or brainstem encephalitis, myelitis, neuropathy, Lambert-Eaton Syndrome
ANNA3		+	+	+	Paraneoplastic cerebellar degeneration combined with limbic or brainstem encephalitis, myelitis, neuropathy
Zic4		+	+	+	Paraneoplastic cerebellar degeneration (mostly lung cancer), mostly isolated, very rarely combined with Lambert-Eaton myasthenic syndrome
Sox1		+	+	+	Paraneoplastic cerebellar degeneration, isolated or combined with brainstem encephalitis neuropathy, Lambert-Eaton syndrome
DNER		+	+	+	Isolated ataxia or combined with encephalopathy or neuropathy
mGluR1		+	+	+	Isolated or combined with dysgeusia, memory or attention deficits, psychiatric problems
GABA_B_R	+	+		+	Isolated or combined with brainstem encephalitis or in encephalitis with opsoclonus, chorea and seizures
GQ1b	+	+		+	Miller-Fisher syndrome with ophthalmoplegia, mydriasis and areflexia
GFAP	+	+		+	Combined in meningoencephalomyelitis (or limited forms) with encephalopathy with epilepsy, cognitive or psychiatric problems, myelopathy (longitudinal or transversal); red flags: meningeal symptoms (headache, photophobia, neck stiffness), optic disk oedema, myelopathy; MRI: frequently characteristic radial linear periventricular or cerebellar gadolinium enhancement
Ca/ARHGAP26		+	+	+	Rare; isolated or combined with hyperekplexia or cognitive decline
Homer-3		+	+	+	Rare; isolated or combined with encephalopathy
ITPR1		+	+		Rare; clinical data scarce
CARP VIII		+	+		Rare; rapidly progressive paraneoplastic cerebellar ataxia
PKC-γ		+	+		Rare; two patients with paraneoplastic cerebellar ataxia
GluR-δ2	+	+	+	+	Rare; isolated or combined with encephalopathy
Nb/AP3B2		+		+	Rare; combined with pyramidal involvement
ATP1A3		+		+	Rare; combined with vertical gaze palsy, spastic tetraparesis, deterioration of visual acuity
**Stiff person spectrum disorders**					
GAD	+	+	+	+	Isolated or combined SPSD e.g. with ataxia, epilepsy, oculomotor disturbance, dysautonomia, pyramidal signs, sensory symptoms or encephalopathy; often associated with organ-specific autoimmunity, e.g. diabetes type 1, vitiligo, thyroiditis, pernicious anaemia
GlyR	+	+	+	+	Isolated or combined SPSD e.g. oculomotor disturbance, bulbar symptoms, dysautonomia, pyramidal signs, sensory symptoms, encephalopathy
Amphiphysin		+	+	+	Isolated or combined with with sensory ganglionopathy, myelopathy
					Paraneoplastic SPSD with breast or small cell lung cancer
GABA_A_R	+	+	+	+	Isolated or combined with epilepsy; partly co-occurring with → GAD antibodies
DPPX	+	+		+	Combined SPSD with prominent hyperekplexia and myoclonus, cerebellar ataxia, dysautonomia, pyramidal signs, sensory symptoms, cognitive problems; red flags: prolonged diarrhoea, other gastrointestinal symptoms
Gephyrin		+		+	Single case, combined SPSD with dysarthria and dysphagia
GlyT2		+	+	+	Preliminary report of two cases, patients were also positive for → GAD antibodies
GABARAP		+	+	+	All reported patients were also positive for → GAD antibodies
Ri		+		+	Combined SPSD as part of brainstem encephalitis
**Paroxysmal dyskinesias**					
LGI1		+	+	+	Characteristic FBDS, isolated or combined with other signs of limbic encephalitis; red flags: hyponatraemia, bradycardia as neurocardiac prodrome
NMDAR	+	+	+	+	Paroxysmal dystonic posturing preceding encephalitis
AQP4	+	+	+	+	Painful tonic spasms in neuromyelitis optica, often combined with sensory, motor, visual or sphincter disturbance
**Neuromyotonia and myokymia**					
CASPR2		+	+	+	Main cause of immune-mediated peripheral nerve hyperexcitability, either in isolation or combined with pain, neuropathy or as part of Morvan syndrome
LGI1		+	+	+	Rarely in CASPR2 antibody-negative cases
**Tics**					
D2R	+		+		Very rare; reported in 4/44 children with Tourette’s syndrome, relevance in clinical practice still to be established
**Tremor**					
MAG		+		+	In chronic inflammatory demyelinating neuropathy
LGI1		+		+	As part of more widespread involvement in encephalitis
CASPR2					As part of more widespread involvement in encephalitis
DPPX	+	+		+	As part of more widespread involvement in encephalitis
NMDAR	+	+		+	As part of more widespread involvement in encephalitis
Yo		+		+	Holmes tremor in cerebellar ataxia
GFAP	+	+		+	Combined in meningoencephalomyelitis (or limited forms) with encephalopathy with epilepsy, cognitive or psychiatric problems, myelopathy (longitudinal or transversal), or ataxia; red flags: meningeal symptoms (headache, photophobia, neck stiffness), optic disk oedema, myelopathy; MRI: frequently characteristic radial linear periventricular or cerebellar gadolinium enhancement
**Sleep movement disorders**					
NMDAR	+	+		+	Status dissociatus and agrypnia excitata in encephalopathic syndrome
CASPR2		+		+	Status dissociatus and agrypnia excitata in Morvan syndrome
GABA_B_R		+		+	Agrypnia excitata in encephalopathic syndrome
Ma2		+		+	RBD in characteristic → parkinsonism syndrome
LGI1		+		+	RBD in limbic encephalitis
DPPX		+		+	Periodic limb movements of sleep
IgLON5		+		+	RBD and non-RBD and parasomnias

We suggest a phenomenological approach that takes into account the main movement disorder presentation, age (childhood versus adulthood) and the occurrence of other symptoms. ‘Isolated’ refers to where the respective movement disorder is the only symptom, whereas ‘combined’ denotes additional signs. For example, in SPSD, stiffness, spasms and hyperekplexia are considered as the core features and would be expected in ‘isolated’ forms. Additional signs like ataxia or epilepsy would be indicated as ‘combined’. Such designations may warrant revision in the future as the spectrum keeps expanding. The right column provides more details about the clinical or radiological phenotype.

This table is aimed as a reference and includes all antibodies for the sake of completeness. However, some antibodies are more frequent than others. Please refer to Table 2 for relative frequency of antibodies in clinical practice.

^a^Frequently no antibody found, and antibodies not syndrome-specific.

ANNA1/2 = anti-neuronal nuclear autoantibody 1/2; CARP VIII = carbonic anhydrase-related protein VIII; CASPR2 = contactin associated protein 2; CRMP5 = collapsin response mediator protein 5; DPPX = dipeptidyl peptidase-like protein 6; D2R = dopamine 2 receptor; FBDS = faciobrachial dystonic seizures; GABA_A_R and GABA_B_R = γ-aminobutyric acid type A and type B receptors; GAD = glutamic acid decarboxylase; GluR-δ2 = glutamate receptor delta 2; GlyR = glycine receptor; GlyT2 = glycine transporter 2; GQ1b = ganglioside Q1b; IgLON5 = IgLON family member 5; mGluR1 = metabotropic glumatate receptor type 1; NMDAR = *N*-methyl-d-aspartate receptor; PKC-γ = protein kinase C gamma; Sox1 = Sry-like high mobility group box protein 1; SPSD = stiff person spectrum disorders; VGCC = voltage gated calcium channel; Zic4 = Zic family member 4.

**Table 2:** Overview: the discussed neuronal antibodies, their relative frequency, target antigens, associated clinical and oncological spectrum

Antibody target	Relative frequency in clinical practice	Tumour association	Movement disorder presentation	Other clinical features
**Neuronal surface antibodies**				
AQP4	++	(+)/−	Painful tonic spasms	Neuromyelitis optica spectrum disorders, typically with optic neuritis, pyramidal weakness, sensory symptoms, bladder disturbance
		Rarely; lung or breast cancer, teratoma		
ATP1A3	Not yet clear (single case report in 2015)	+	Cerebellar ataxia	Vertical gaze palsy, spastic tetraparesis, deterioration of visual acuity
		Colon adenocarcinoma		
CASPR2	++	+/−	Cerebellar ataxia, chorea, neuromyotonia, myokymia	Morvan syndrome, limbic encephalitis, neuropathy (rarely Guillain-Barré-like syndrome), neuropathic pain
		In ∼20%: thymoma ≫ lung, prostate, sigmoid or thyroid cancer, myeloma		
DNER	+	+++	Cerebellar ataxia	Encephalitis, neuropathy
		In∼ 90%: Hodgkin lymphoma ≫ lung carcinoma		
DPPX	++	+/−	SPSD, myoclonus, startle, ataxia, tremor, parkinsonism, opsoclonus myoclonus	Multifocal encephalitis or brainstem encephalitis with prominent gastrointestinal symptoms (prolonged diarrhoea, constipation), other dysautonomic signs (urinary or erectile dysfunction, cardiac arrhythmia, thermodysregulation, Raynaud’s phenomenon), sensory disturbance (allodynia, paraesthesia)
		In ∼7%: B-cell neoplasms		
D2R	Very rare	−	Basal ganglia encephalitis in children with dystonia, chorea or parkinsonism; Sydenham’s chorea	Psychiatric and sleep disturbance
GABA_A_R	++	+/−	Chorea, dystonia or ataxia (as part of a more widespread encephalopathy), opsoclonus myoclonus syndrome; possible association with SPSD	Encephalopathy with epilepsy, behavioural or cognitive problems or reduced consciousness; frequent multifocal T_2_ hyperintensities on MRI; tendency to autoimmune predisposition (coexisting antibodies, e.g. GAD or NMDAR antibodies, thyroid autoimmunity, idiopathic thrombocytopenic purpura, gluten sensitivity or myasthenia)
		In ∼40%: thymoma, lung carcinoma, rectal cancer, myeloma		
GABA_B_R	++	+/−	Opsoclonus myoclonus ataxia syndrome, cerebellar ataxia	Limbic encephalitis with prominent seizures
		In ∼60%: small cell lung cancer ≫ breast cancer multiple myeloma, rectal carcinoma, oesophageal carcinoma		
GluRδ2	Very rare; case reports from Japan only	Para/post-infectious	Cerebellar ataxia	(Limbic) encephalitis, epilepsy
GlyR	+++	+/−	SPSD, myoclonus, hyperekplexia, ataxia	Brainstem encephalitis; reported also in: optic neuritis; limbic / epileptic encephalopathy, epilepsy, steroid-responsive deafness (clinical relevance less clear)
		In ∼9%: thymoma > small cell lung cancer, breast cancer, Hodgkin lymphoma, chronic lymphocytic leukaemia		
GlyT2	Not yet clear; preliminary report of two patients		SPSD	Co-occurring with → GAD antibodies
IgLON5	+		Gait instability, cerebellar ataxia, chorea in patients with tau brain pathology	REM and Non-REM sleep behaviour disorder; sleep apnoea, stridor, dysphagia, oculomotor disturbance, cognitive decline, dysautonomia
LGI1	+++	(+)/− In ∼ 7%: liver carcinoid, neuroendocrine pancreas tumour, mesothelioma, rectal carcinoma	Faciobrachial dystonic seizures, chorea, parkinsonism	Limbic encephalitis; hyponatraemia, bradycardia


mGluR1	+	+/−	Cerebellar ataxia	Memory or attention deficits, dysgeusia, psychiatric problems (auditory hallucinations, paranoia)
		In ∼ 43%: Hodgkin lymphoma ≫ prostate adenocarcinoma		
NMDAR	++++	+/−	Orofacial and limb dyskinesia, chorea, dystonia, myoclonus, ataxia, parkinsonism, paroxysmal dyskinesias	Prodromal infectious-like symptoms, neuropsychiatric disturbance, encephalopathy with epilepsy, cognitive deficits, reduced consciousness, dysautonomia, central hypoventilation
		In ∼40%: ovarian teratoma ≫ extraovarian teratomas, ovarian carcinomas; lung, breast, testicular and pancreatic tumours		
Neurexin-3α	+		Mild orofacial dyskinesias	Encephalopathy with epilepsy, reduced consciousness, memory deficits, psychomotor agitation
VGCC	++	++	Cerebellar ataxia	Lambert-Eaton myasthenic syndrome, encephalopathy, neuropathy
		Tumour association varies in different studies between 20 and 90%, mostly small cell lung cancer		
VGKC_complex_^a^;	N/A	N/A	N/A	N/A
**Antibodies targeting intracellular, synaptic proteins**					
Amphiphysin	++	+++	SPSD	Sensory ganglionopathy, myeolpathy
		Breast cancer, small cell lung cancer		
GAD	++++	+/−	SPSD, cerebellar ataxia	Limbic encephalitis; focal epilepsy; often concomitant autoimmunity (e.g. diabetes type 1, thyroid disease, vitiligo, pernicious anaemia)
		(rarely, various tumours)		
Gephyrin	Single case	(+)	SPSD	–
		Mediastinal carcinoma		
GABARAP	+	−	SPSD	Only in association with → GAD antibodies
**Antibodies targeting cytoplasmic and nuclear antigens**					
ANNA3^b^	+	+++	Cerebellar ataxia	Sensory/sensorimotor neuropathy, myelopathy, brainstem or limbic encephalitis
		Small cell lung cancer, lung or oesophageal adenocarcinoma		
AP3B2/Nb	Single case	−	Cerebellar ataxia	Pyramidal tract involvement
ARHGAP26/ Ca	Very rare; six cases	+/−	Cerebellar ataxia	Limbic encephalitis
		Ovarian carcinoma		
CARP VIII	Very rare; two cases	++	Cerebellar ataxia	−
		Ovarian carcinoma, melanoma		
CRMP5/CV2	++	+++	Chorea	Optic neuritis, myelitis (can mimic neuromyelitis optica), cognitive decline, neuropathy
		Small cell lung cancer, thymoma		
GFAP	+	+/− In ∼34%: prostate and gastroesophageal adenocarcinomas, myeloma, melanoma, colonic carcinoid, parotid pleomorphic adenoma, teratoma	Cerbebellar ataxia, tremor, undefined movement disorders	Menigeoencephalomyelitis or limited forms, with headache, cognitive problems, optic papillitis, sensory disturbance, gastrointestinal and urogenital dysautonomia, neuropathy; often concomitant autoimmunity (e.g. diabetes type 1, thyroid disease, myasthenia, rheumatoid arthritis, alopecia)

Homer-3	Very rare; four cases	−	Cerebellar ataxia	Epilepsy, confusion

Hu / ANNA-1	+++	+++	Chorea, cerebellar ataxia, opsoclonus myoclonus ataxia syndrome	Encephalomyelitis, limbic encephalitis, brainstem encephalitis, sensory neuropathy, gastrointestinal pseudoobstruction
		Small cell lung cancer ≫ neuroblastoma or intestinal, prostate, breast, bladder, and ovary carcinoma		
ITPR1	+	+++	Cerebellar ataxia	Peripheral neuropathy
		Breast cancer associated with *BRCA1*		
Ma2/Ta	++	+++	Parkinsonism	Limbic, diencephalic or brainstem encephalitis, myelopathy or radiculoplexopathy, with encephalopathy, hypothalamic-pituitary endocrine dysfunction, weight gain, prominent sleep disorders, eye movement abnormalities (opsoclonus, supranuclear gaze palsy), dysphagia, muscular atrophy, fasciculations
		Testis ≫ lung cancer; rarely no neoplasia		
Ri / ANNA-2	++	+++	Dystonia (jaw closing dystonia, laryngospasms), opsoclonus myoclonus ataxia syndrome, oculopalatal myoclonus, cerebellar ataxia, SPSD	Brainstem encephalitis with cranial nerve palsies, nystagmus, dysarthria, ataxia, rigidity, trismus, pyramidal signs
		Gynaecological tumours, mainly breast cancer, and lung cancer		
Sox1	++	+++	Cerebellar ataxia	Lambert-Eaton myasthenic syndrome, sensory/sensorimotor neuropathy, brainstem encephalitis
		Lung cancer		
Yo/PCA1	+++	+++	Cerebellar ataxia	Rhombencephalitis, peripheral neuropathy
		Gynaecological tumours		
PKCγ	Very rare; two cases	++	Cerebellar ataxia	–
		Non-small cell lung cancer, hepatobiliary adenocarcinoma		
Zic4	+++	+++	Cerebellar ataxia	–
		Small cell lung cancer ≫ ovarian adenocarcinoma		

Of note, exact prevalences are unknown, and relative frequencies are based on the literature and own experience only, but given to indicate their relevance in clinical practice.

^a^Antibodies against the voltage gated potassium channel complex (VGKC_complex_) were previously detected by radioimmunoassay (RIA), which does not allow distinction of the later identified, specific targets (LGI1, CASPR2, or very rarely contactin-2, or possibly some yet uncharacterized antigens). To test specifically for CASPR2 or LGI1 antibodies, cell-based assays are applied, and this may yield positive results even if the VGKC_complex_-RIA has been negative. Conversely, there is a proportion of sera positive in the VGKC_complex_-RIA that do not harbour antibodies that recognize LGI1 and/or CASPR2, and it has been argued this is unlikely to indicate true autoimmune disease.

^b^Antigen unknown.

+ = rare; ++ = occasional; +++ = frequent; ++++ = very frequent; CARP VIII = carbonic anhydrase VIII; CASPR2 = contactin-associated protein 2; CRMP5/CV2 = collapsin response mediator protein 5; D2R = dopamine 2 receptor; DPPX = dipeptidyl peptidase-like protein 6; GABAAR = γ-aminobutyric acid A receptor; GABABR = γ-aminobutyric acid B receptor; GAD = glutamic acid decarboxylase; GluRδ2 = glutamate receptor delta 2; GlyR = glycine receptor; GlyT2 = glycine transporter 2; Hu/ANNA-1 = Hu proteins (HuD, HuC)/anti-neuronal nuclear autoantibody 1; IgLON5 = IgLON family member 5; Ma2/Ta = PNMA2; mGluR1 = metabotropic glutamate receptor 1; NMDAR = *N*-methyl-d-aspartate receptor; PKCγ = protein kinase C gamma; Ri/ANNA-2 = Nova-1, Nova-2/anti-neuronal nuclear autoantibody 2; Sox1 = Sry-like high mobility group box protein 1; SPSD = stiff person spectrum disorders; VGCC = P/Q-type voltage gated calcium channel; VGKC_complex_ = voltage gated potassium channel complex a; Yo/PCA1 = CDR62/ CDR2, CDR34/ CDR1; Zic4 = Zinc finger protein 4.

### Chorea and dyskinesias

Chorea is characterized by brief, irregular, purposeless movements that unpredictably flit from one body part to another (Edwards *et al.*, 2016). Chorea occurs as the sole or main feature of various conditions, which may broadly be divided into inherited (most commonly Huntington’s disease), and acquired causes, including autoimmune chorea. Sydenham’s chorea and chorea in antiphospholipid syndrome or systemic lupus erythematosus are prime examples of the latter, but autoimmune chorea and dyskinesias also occur with a number of neuronal antibodies in children and adults (Table 1).

Distinct dyskinesias, which affect mainly the mouth and the limbs and persist in states of decreased responsiveness, are characteristic for the encephalitis associated with NMDAR antibodies (Dalmau *et al.*, 2008; Titulaer *et al.*, 2013). Of note, a similar picture with a prodromal phase with fever and headache, and evolution to neuropsychiatric disturbance with subsequent dysautonomia, mild orofacial dyskinesias, decreased consciousness, and seizures can occur also with the newly discovered neurexin-3α antibodies (Gresa-Arribas *et al.*, 2016). NMDAR-antibody encephalitis typically manifests in an age-dependent manner: adults tend to present with neuropsychiatric disturbance and behavioural problems initially, while in children, epilepsy and movement disorders, such as chorea, are more prominent. Children with NMDAR-antibody encephalitis may be misdiagnosed as having Sydenham’s chorea, particularly in early stages of the disease, as both disorders feature a subacute onset and prominent behavioural/neuropsychiatric disturbances (Hacohen *et al.*, 2014; Udani *et al.*, 2016). Overall, isolated movement disorder presentations are, however, extremely rare in NMDAR-antibody-related encephalitis, and typically, the presence of seizures, dysautonomia, or ataxia should alert the neurologist to request testing for NMDAR antibodies (Titulaer *et al.*, 2013). Another red flag is preceding herpes simplex virus encephalitis (HSVE). HSVE can trigger CNS autoimmunity, and the well-recognized choreic, ballistic or athetoid relapses that sometimes follow HSVE by 2–6 weeks are associated with NMDAR antibodies (Armangue *et al.*, 2014*a*). Furthermore, chorea is seen also with antibodies against the striatal dopamine receptor 2 (D2R antibodies). D2R antibodies have only been reported in children, either with basal ganglia encephalitis, Sydenham’s chorea or in choreoathetoid relapses after HSVE (Dale *et al.*, 2012; Mohammad *et al.*, 2014*b*). They are very rare in routine clinical testing (personal experience, Irani, Vincent and Waters).

Later in adulthood, paraneoplastic chorea comes into the differential diagnosis. It occurs mainly in association with CRMP5 or Hu antibodies, typically combined with other neurological signs, and features on MRI characteristic fluid-attenuated inversion recovery (FLAIR) hyperintensities in the white matter, basal ganglia, and medial temporal lobes (Vigliani *et al.*, 2011). Sometimes, however, these red flags are lacking and paraneoplastic chorea may resemble Huntington’s disease, including caudate atrophy on MRI (Vigliani *et al.*, 2011).

Finally, LGI1 or CASPR2 antibodies can also cause isolated or combined chorea/hemichorea, with or without neuropsychiatric symptoms, but typically without any underlying malignancy (Tofaris *et al.*, 2012; O’Toole *et al.*, 2013). Akin to the patients with NMDAR antibodies, over time these patients often develop a more typical encephalopathy with multiple clinical features.

### Dystonia

Dystonia (sustained or intermittent muscle contractions causing abnormal movements or postures) is the only sign in primary/isolated dystonias, but associated with other symptoms in a variety of conditions such as heredodegenerative, metabolic, infectious, and autoimmune disorders (Edwards *et al.*, 2016). Antibody-related dystonia does not mimic primary dystonia, which follows a characteristic pattern in its anatomical distribution and age at onset (Edwards *et al.*, 2016). For example, young-onset primary dystonia features typically limb onset with subsquent generalization. In contrast, there are a few reports of children and young adults, who harboured NMDAR antibodies and had hemidystonia or craniocervical dystonia as the most prominent feature (Rubio-Agusti *et al.*, 2011; Mohammad *et al.*, 2014*a*). More often, antibody-related dystonia is one symptom in an encephalopathic syndrome associated with various antibodies (Table 1) (Dalmau *et al.*, 2004; Dale *et al.*, 2012). Thus, autoimmune encephalitis, particularly in children, comes into the differential diagnosis of encephalopathies with dystonia, such as mitochondrial or neurometabolic disease, and potentially treatable disorders like biotin-responsive dystonia or DOPA synthesis pathway disorders (Edwards *et al.*, 2016). Of note, patients with NMDAR-antibodies often have oculogyric crises akin to children with dopamine-synthesis disorders. In adults, jaw-closing dystonia with recurrent episodes of laryngospasm is an important syndrome and pathognomonic for paraneoplastic brainstem encephalitis with Ri antibodies (Pittock *et al.*, 2010). The symptoms can be severe enough to impair nutrition or require prophylactic tracheostomy. The MRI may be unrevealing in some cases, or display T_2_ hyperintensities mainly in the pons and temporal lobes (Pittock *et al.*, 2010). An important differential diagnosis is ‘lockjaw’, resembling tetanus, as seen in stiff person spectrum disorders (SPSD) with glycine receptor antibodies (Doppler *et al.*, 2016).

### Myoclonus

Myoclonus (very brief, shock-like jerks) can be a feature of many underlying aetiologies. Subacute-onset of myoclonus with encephalopathy will invoke a wide differential diagnosis including metabolic (e.g. renal, liver failure), toxic (e.g. lead, lithium) and infectious (e.g. prion disease, Whipple’s disease) processes, and autoimmune encephalitis. However, isolated myoclonus is rarely seen with neuronal autoantibodies (McKeon *et al.*, 2007). Myoclonus is a striking feature in patients with encephalitis with DPPX antibodies, who often have prodromal, prolonged diarrhoea with weight loss and other signs of dysautonomia (Boronat *et al.*, 2013; Tobin *et al.*, 2014). More frequently, however, myoclonus has been reported in LGI1- and CASPR2-antibody-associated encephalitis, an important mimic of Creutzfeldt-Jakob disease (CJD) (Geschwind *et al.*, 2008; Tan *et al.*, 2008). The MRI with FLAIR/diffusion-weighted imaging hyperintensities of the basal ganglia and the cortical ribbon sign as seen in CJD can also be present in some patients with LGI1 antibodies, and in both conditions, the basic CSF parameters are often normal (Geschwind *et al.*, 2008; Vitali *et al.*, 2011). Red flags pointing towards LGI1 antibodies are seizures, including faciobrachial-dystonic seizures (see below, may themselves account for many descriptions of myoclonus), episodic bradycardia, and serum hyponatremia (Naasan *et al.*, 2014).

Predominant myoclonus of the legs, affecting stance and gait, is an emerging phenotype associated with CASPR2 antibodies and may have been noted in previous reports, which lacked antibody subtyping (Hegde *et al.*, 2011; Krogias *et al.*, 2013; Govert *et al.*, 2016). The patients were middle-aged or elderly males, with additional neuropathic pain, fasciculations or cognitive impairment, who responded promptly to immunotherapy.

Myoclonus is a major feature in progressive encephalomyelitis with rigidity and myoclonus (see below), and one of the defining characteristic of opsoclonus-myoclonus syndrome (OMS). Neuronal antibodies are identified only in approximately a third of patients with OMS (Armangue *et al.*, 2016) and their variety (Table 1) and lack of syndrome-specificity indicate that they are probably only an epiphenomenon of a wider autoimmune process, which may be postinfectious (e.g. seroconversion of HIV) (Klaas *et al.*, 2012), or paraneoplastic. In children, OMS is frequently associated with neuroblastoma and typically presents in the first 3 years of life. In adolescents, there is a subgroup of patients with additional brainstem signs, who have ovarian teratomas (without NMDAR antibodies), and who respond well to immunotherapy (Armangue *et al.*, 2014*b*). Older age and encephalopathy associate with paraneoplastic OMS (cancer of lung and breast prevailing) with a poorer outcome (Armangue *et al.*, 2016).

Myoclonus can also be a feature of ‘steroid responsive encephalopathy with thyroid antibodies’ (SREAT) and gluten sensitivity-related neurological disease (Edwards *et al.*, 2016). The associated thyroid, gliadin or tissue transglutaminase antibodies indicate an autoimmune predisposition but are unlikely to themselves cause the neurological manifestation, as they do not target the extracellular domain of neuronal proteins. However, GABA_A_R antibodies characterize an encephalitis with prominent epilepsy, cortical and subcortical hyperintensities on T_2_-weighted MRI, and a strong association with thyroid autoimmunity: thus, some of these patients were likely previously termed SREAT (Petit-Pedrol *et al.*, 2014). Indeed, other SREAT cases have co-existent neuronal surface antibodies and likely will benefit from more careful future classifications (Tuzun *et al.*, 2011). Similarly, neuronal antibodies co-occurring in gluten-related disease with myoclonus and ataxia may account for some of the associated neurological manifestations (McKeon *et al.*, 2014; Petit-Pedrol *et al.*, 2014).

### Paroxysmal dyskinesias

The primary paroxysmal dyskinesias are a group of rare, genetically determined inherited disorders typified by brief self-limiting attacks of involuntary movements (Edwards *et al.*, 2016). There is frequently a positive family history of autosomal dominant inheritance, and, most importantly, they all manifest early in life, typically in adolescence. Three phenotypes are defined by the duration of attacks and particular triggers: paroxysmal kinesigenic (attacks lasting seconds to minutes, precipitated by sudden movement; mostly caused by PRRT2 mutations), non-kinesigenic (attacks lasting minutes to hours, triggered by alcohol, coffee or fatigue; mostly caused by myofibrillogenesis regulator 1 mutations), and exercise-induced dyskinesias (gradual onset of dystonia after prolonged exercise; mostly caused by SLC2A1 mutations).

By contrast, the prototypical antibody-associated paroxysmal dyskinesias are faciobrachial dystonic seizures (FBDS) and usually manifest late in life (median around 66 years, range 28–92) (Irani *et al.*, 2011, 2013). Their phenotype is distinctive, with brief (typically <3 s), and frequent episodes (up to several hundred per day) of stereotypical dystonic posturing (Supplementary Video 1). These mainly involve face, arm or leg, or combinations of these, and usually involve one side at a time, although the affected side might alternate in an individual. FBDS occur spontaneously or may be triggered by high emotions, auditory stimuli or movement (Irani *et al.*, 2011, 2013). A longer duration, simultaneous bilateral involvement, and FBDS as a cause of drop attacks are other recognized clinical manifestations (Irani *et al.*, 2011, 2013). FBDS are consistently associated with LGI1 antibodies. In the primary paroxysmal dyskinesias, there has been historical debate as to whether they represent a movement disorder or epilepsy, and similar arguments can be applied to FBDS. Signs indicative of an epilepsy include prominent automatisms, sensory aura, and post-ictal fear and speech arrest, but only few patients show clear ictal epileptiform discharges on EEG (Irani *et al.*, 2011). On the other hand, ∼40% of patients’ MRIs show basal ganglia hyperintensities on T_1_- or T_2_-weighted sequences (Irani *et al.*, 2013; Flanagan *et al.*, 2015). FBDS appear to respond preferentially to immunotherapy, and it is hypothesized that timely treatment prevents the development of cognitive impairment associated with limbic encephalitis (Irani *et al.*, 2013).

Brief dystonic episodes without EEG correlate and paroxysmal exercise-induced foot weakness were also reported in single cases with NMDAR antibodies (Xia and Dubeau, 2011; Labate *et al.*, 2013). Prominent pain is not a feature of primary paroxysmal dyskinesias, but typical for the tonic spasms associated with demyelinating disease, and interestingly these occur more commonly with AQP4 antibodies than in multiple sclerosis (Kim *et al.*, 2012).

### Parkinsonism

Parkinsonism, defined by bradykinesia, is of course the hallmark feature of idiopathic Parkinson’s disease, which often also shows unilateral onset and persistent asymmetry, rest tremor (typically ‘pill-rolling’), and an excellent response to l-DOPA. In contrast, ‘atypical parkinsonism’ is defined by features not in keeping with idiopathic Parkinson’s disease and typically by a poor response to l-DOPA. It has various aetiologies, mostly neurodegenerative diseases including progressive supranuclear palsy (PSP), corticobasal degeneration, or multisystem atrophy, and, less frequently, infectious (flavivirus, HIV, Whipple’s disease, prion disease), toxic or metabolic causes. Paraneoplastic parkinsonism is another rare, but important differential diagnosis and has been described in association with CRMP5, Ri, and Ma2-antibodies (Yu *et al.*, 2001; Pittock *et al.*, 2003; Dalmau *et al.*, 2004). A rapidly progressive, disabling disease course is a red flag, but not always present (Yap *et al.*, 2017). Parkinsonism with Ma2 antibodies has a characteristic PSP-like phenotype with supranuclear gaze palsy (vertical > horizontal) and eye closure resembling apraxia of lid opening (Dalmau *et al.*, 2004). Distinctive features are hypothalamic-pituitary dysfunction, weight gain, and prominent sleep disorders including excessive daytime sleepiness, rapid eye movement (REM) sleep behaviour disorder (RBD), and narcolepsy-cataplexy (Dalmau *et al.*, 2004; Compta *et al.*, 2007). Ma2 antibodies associate with limbic, diencephalic and brainstem encephalitis, myelopathy and radiculoplexopathy, thus providing further clinical signs that are suggestive of this entity (Dalmau *et al.*, 2004). The typical MRI pattern of Ma2-antibody encephalitis are thalamic and hypothalamic T_2_ hyperintensities, whereas basal ganglia involvement is more often seen with CRMP5 antibodies (Dalmau *et al.*, 2004). Paraneoplastic parkinsonism can also manifest as corticobasal syndrome, sometimes without an identifiable antibody but with striking hyperintensities on T_2_-weighted MRI (McKeon *et al.*, 2009). However, non-paraneoplastic encephalitides also can manifest with parkinsonism: indeed, patients with LGI1, DPPX and GAD antibodies have been misdiagnosed with Parkinson’s disease, PSP or multisystem atrophy (Pittock *et al.*, 2006; Tobin *et al.*, 2014; Kurtis *et al.*, 2015). In children with acquired parkinsonism, the work-up should also include testing for NMDAR and D2R antibodies (Dale *et al.*, 2012; Mohammad *et al.*, 2014*a*).

### Cerebellar ataxia

Idiopathic or paraneoplastic autoimmunity is an important aetiology of ataxia, where age, tempo of disease progression, and associated signs dictate the differential diagnosis. The most frequently identified autoimmune ataxia is associated with GAD antibodies and is often accompanied by other autoimmune disorders (diabetes, thyroid disease, pernicious anaemia, vitiligo) (Arino *et al.*, 2014). It can present with a slowly progressive course or subacutely, with either isolated cerebellar signs or additional signs such as pyramidal tract involvement or features of stiff person syndrome. Often, there is up- or downbeat nystagmus. Episodes of brainstem or cerebellar dysfunction are a red flag, as they precede the chronic course in one-third of patients, and enter the differential diagnosis of episodic ataxia type 2 (Arino *et al.*, 2014).

A similar phenotype, with early vertigo or ataxia episodes and concomitant autoimmunity, can also be seen in ataxia with coeliac disease or gluten-related ataxia, often with additional pyramidal signs or neuropathy. The pathophysiology and the role of neuronal autoantibodies in this entity are unclear (McKeon *et al.*, 2014), but DPPX antibodies would clearly come into the differential diagnosis of ataxia and prolonged diarrhoea (Boronat *et al.*, 2013; Balint *et al.*, 2014*a*; Tobin *et al.*, 2014).

A subacute onset of ataxia with progression over weeks to months and severe disability is often seen with paraneoplastic cerebellar degeneration (PCD). PCD associates with almost all of the classical onconeuronal antibodies (Table 1) (Shams’ili *et al.*, 2003). A pure cerebellar syndrome occurs classically in females with gynaecological tumours and Yo antibodies, or in males with Hodgkin lymphoma and DNER antibodies (Shams’ili *et al.*, 2003; de Graaff *et al.*, 2012). Further neurological signs in addition to the ataxia (Table 2) are, however, frequent, and may guide a syndromic diagnosis: for example, ataxia and proximal muscle weakness is seen in Lambert Eaton myasthenic syndrome with VGCC antibodies, typically with lung cancer.

Combined phenotypes of cerebellar ataxia with encephalopathy and/or brainstem dysfunction are also seen with several of the newer antibodies, e.g. against GABA_B_R, CASPR2 and GFAP (Becker *et al.*, 2012; Jarius *et al.*, 2013; Balint *et al.*, 2014*a*; Fang *et al.*, 2016; Flanagan *et al.*, 2017). Ataxia occurs also in NMDAR-antibody encephalitis in children, but only rarely in adults (Titulaer *et al.*, 2013). Neuropathy, areflexia and ophthalmoplegia are the characteristic accompaniments of cerebellar-like ataxia in Miller-Fisher syndrome with GQ1b antibodies (Yuki *et al.*, 1993). Lastly, a group of rarer autoantibodies, which target proteins also affected by mutations in genetic ataxias, are discussed below (‘Pathophysiology and genetic parallels’ section).

### Stiff person spectrum disorders and acquired hyperekplexia

SPSD are characterized by the core symptoms of fluctuating muscle stiffness with superimposed spasms, and an exaggerated startle response (hyperekplexia). The manifestations include classical stiff person syndrome, stiff limb syndrome, and variants combined with additional neurological symptoms (stiff person plus) or with a potentially fatal disease course in progressive encephalomyelitis with rigidity and myoclonus (PERM). Acquired hyperekplexia also enters this spectrum of disorders. In practice, SPSDs are still often misdiagnosed and symptoms mistaken for psychogenic, dystonic posturing, or related to parkinsonism (Balint *et al.*, 2014*b*). Nevertheless, stiffness and spasms can cause significant morbidity with falls, fractures, or even death due to respiratory failure.

The most frequent antibodies remain those against GAD and the glycine receptor (GlyR), and less frequently, amphiphysin (Balint *et al.*, 2015; Martinez-Hernandez *et al.*, 2016). The antibody spectrum associated with SPSD has expanded with recent reports of DPPX, GABA_A_R and GlyT2 antibodies, but further work is needed to elucidate each of their roles in SPSD (Balint and Bhatia, 2016). Overall, it is difficult to predict the antibody specificity based on clinical grounds, as there is significant overlap between the various antibodies with regard phenotype and disease course. However, patients with GAD antibodies often also have cerebellar ataxia and, less frequently, temporal lobe epilepsy (Balint and Bhatia, 2016; Martinez-Hernandez *et al.*, 2016). Myelopathy and sensory neuropathy, in association with SPSD strongly indicate a paraneoplastic syndrome with amphiphysin antibodies (Murinson and Guarnaccia, 2008).

GlyR antibodies associate with prominent brainstem involvement including oculomotor or bulbar disturbance, myoclonus and hyperekplexia, and often sensory and autonomic symptoms (Martinez-Hernandez *et al.*, 2016). Patients with DPPX antibodies tend to have trunk stiffness, prominent cerebellar ataxia and striking hyperekplexia, together with various degrees of dysautonomia, somatosensory disturbances, and cognitive decline (Balint *et al.*, 2014*a*). Gastrointestinal hyper- or hypomobility and marked weight loss are strong indicators of DPPX antibodies (Tobin *et al.*, 2014).

### Tics

Tics are rapid, brief, stereotyped movements or vocalizations. Eye blinking, shoulder shrugging, grimacing, sniffing or grunting are examples of ‘simple motor or vocal tics’, whereas ‘complex tics’ designate sequences of stereotyped movements, or words or phrases (Edwards *et al.*, 2016). Typically, tics wax and wane, and are (temporarily) suppressible, but patients will describe an inner rising tension or anxiety to allow the tics to emerge (premonitory urge). Tics mostly occur as primary disorders during childhood, without associated neurological features. They are also seen as part of the spectrum of paediatric autoimmune neuropsychiatric disorders associated with streptococcal infections (PANDAS). Although it has been speculated that neuronal antibodies may play a role in PANDAS, reproducible evidence for this is lacking. So far, one group has found D2R antibodies in 4 of 44 children with Tourette’s syndrome but not in PANDAS (Dale *et al.*, 2012). Other reports of D2R antibodies in PANDAS are based on methods that are less suitable to detect potentially pathogenic antibodies against native neuronal surface antigens (Morris-Berry *et al.*, 2013). Overall, it appears that D2R antibodies are very rare and not mandatory for the routine diagnostic work-up of tic disorders.

### Tremor

Tremor is defined as a rhythmic, oscillatory movement, usually due to alternate activation of agonist and antagonist muscles (Edwards *et al.*, 2016). Tremor has not been described as an isolated manifestation in antibody-mediated disorders, but can be part of a wider encephalopathic picture in association with LGI1/CASPR2, NMDAR and DPPX antibodies (Tan *et al.*, 2008; Boronat *et al.*, 2013; Mohammad *et al.*, 2014*a*, *b*; Tobin *et al.*, 2014) Similarly, tremor is often part of the presentation of meningoencephalomyelitis (or limited forms) with GFAP antibodies, frequently featuring a characteristic MRI with radial linear periventricular or cerebellar gadolinium enhancement (Fang *et al.*, 2016; Flanagan *et al.*, 2017).

Intention and action tremor or titubation can occur as part of an antibody-related cerebellar syndrome, and Holmes tremor has been described in patients with cerebellar degeneration and Yo antibodies (Peterson *et al.*, 1992). Although Holmes tremor is classically associated with Wilson’s disease and with midbrain lesions, the salient cerebellar ataxia and the atrophy on imaging would argue against such differential diagnoses. Although beyond the scope of this review, tremor may be prominent in chronic inflammatory demyelinating neuropathies, such as those with antibodies against myelin-associated glycoprotein (Edwards *et al.*, 2016).

### Peripheral nerve hyperexcitability: neuromyotonia and myokymia

Peripheral nerve hyperexcitability comprises a spectrum of disorders, such as neuromyotonia, myokymia, or fasciculations, characterized by spontaneous muscle activity and hyperexcitability of motor nerves (Edwards *et al.*, 2016). They are included in this review as they may be confused with movement disorders and are therefore worth keeping in mind in the differential diagnosis. CASPR2 antibodies associate with peripheral nerve hyperexcitability either in isolated neuromyotonia (Isaac’s syndrome) or as part of Morvan’s fibrillary chorea; neuropathic pain is less well recognized but may also be responsive to immunotherapies (Irani *et al.*, 2010, 2012; Klein *et al.*, 2012). LGI1 antibodies are infrequently identified in patients with peripheral nerve hyperexcitability (Irani *et al.*, 2010; Klein *et al.*, 2013).

### Sleep behaviour disorders

RBD is classically seen in α-synuclein-related parkinsonian conditions, and may precede the motor symptoms. The mechanisms of RBD itself remain unclear, but it seems to be caused by dysfunction of certain brainstem structures like the subceruleus and magnocellularis nuclei and their connections, including the amygdala (Iranzo *et al.*, 2016). This may explain RBD as a feature of Ma2 encephalitis, which affects limbic, diencephalic and brainstem structures, or in LGI1-antibody-associated limbic encephalitis (Iranzo *et al.*, 2006; Compta *et al.*, 2007; Irani *et al.*, 2010). Both RBD and non-RBD can be seen in IgLON5-antibody linked neurodegeneration (see below) (Sabater *et al.*, 2014). Status dissociatus (breakdown of the boundaries of the different states of being, which are wakefulness, REM sleep, and non-REM sleep, with motor hyperactivity), and agrypnia excitata (insomnia, motor and autonomic hyperactivation) are a hallmark feature of Morvan syndrome (with CASPR2 antibodies, and less commonly, LGI1 antibodies), but can also be present in encephalitis with NMDAR antibodies or GABA_B_R antibodies (Frisullo *et al.*, 2007; Provini *et al.*, 2011; Stamelou *et al.*, 2012; Abgrall *et al.*, 2015). Finally, a variety of sleep disorders including periodic limb movement and ambiguous sleep are observed with DPPX antibodies (Tobin *et al.*, 2014)

### Approach to antibody testing

Suspicion of an autoantibody-associated disorder may arise because of rapid syndrome evolution, the detailed clinical characteristics, a propensity to autoimmunity in the patient or their family, or a history of a neoplastic process. Further clues may come from inflammatory CSF or MRI findings in the absence of infection. However, autoantibodies may be present even without evidence of inflammation.

When testing for neuronal autoantibodies, we suggest panels based on the predominant movement disorder presentation, age of onset, and the presence or absence of other neurological signs as listed in Table 1. Relative frequencies of autoantibodies as well as further clinical details and possible tumour associations are found in Table 2.

**Figure 1:**
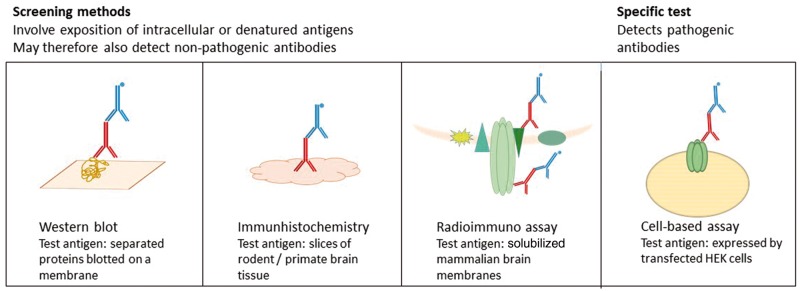
**The different test systems for antibody detection.** HEK = human embryonic kidney cell.

Various assays are used to detect antibodies, with different advantages and shortcomings (Fig. 1). Screening procedures include indirect immunofluorescence or immunohistochemistry, based on slices of rodent brain tissue and western blot, where separated denatured proteins are detected. Often, these require confirmation in more specific test systems like cell-based assays, which overexpress the antigen of interest. The *in vivo* situation, however, is only mimicked by cell-based assays using live cells; in contrast, cell-based assays applying permeabilized or fixed cells may also detect antibodies that are directed against intracellular antigens or non-pathogenic epitopes modified by fixation. Currently, practice varies significantly between laboratories, partly as costs play an inevitable role. Ideally, multi-laboratory assay comparisons are required to understand the relative merits of these tests in different hands.

Similarly, the specimen used may play a role. Some antibodies are primarily detected in the serum, as for example AQP4 antibodies (Jarius *et al.*, 2010), whereas other antibodies may be positive in CSF only, as for example GlyR or NMDAR antibodies (Carvajal-Gonzalez *et al.*, 2014; Gresa-Arribas *et al.*, 2014). This may be due to the lower background interference of CSF compared to serum or due to a predominance of intrathecal antibody synthesis in some disorders. Overall, sensitivity and specificity are highest when both serum and CSF are tested.

## Pathophysiological considerations and the emerging overlap with genetic and degenerative movement disorders

### Pathophysiological considerations and genetic parallels

**Figure 2:**
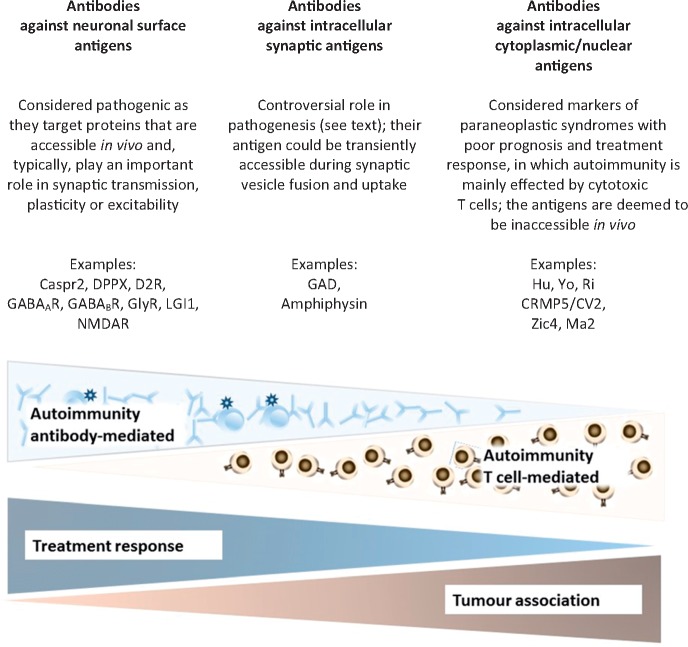
**The three groups of neuronal antibodies and their pathogenic roles, examples, treatment responses and tumour associations.** AMPAR = α-amino-3-hydroxy-5-methyl-4-isoxazolepropionic acid receptor; CASPR2 = contactin associated protein like 2; D2R = dopamine 2 receptor; DPPX = dipeptidyl peptidase like protein 6; GABA_A_R and GABA_B_R = gamma aminobutyric acid type A and type B receptors; GlyR = glycine receptor; LGI1 = leucine rich glioma inactivated protein 1; NMDAR = *N*-methyl-d-aspartate receptor.

Neuronal autoantibodies are neither perfectly specific biomarkers (Box 1) nor necessarily pathogenic, and the exact pathomechanisms leading to specific movement disorder presentations are largely unknown. However, they may be categorized into three groups based on the location of their antigen and its accessibility *in vivo*, and their presumed pathogenic relevance (Fig. 2) (Lancaster and Dalmau, 2012). Evidence for pathogenic relevance comes from observations such as tight correlations between serum or CSF antibody titres and the disease course, pathological studies, and from *in vitro* or *in vivo* experiments. Likewise, phenotypic overlaps with pharmacological modulation or genetic disruption of the antigen can support autoantibody pathogenicity. In the following section, we will discuss the pathogenic role of some of the most relevant neuronal autoantibodies with a focus on parallels between genetic and autoimmune conditions, and the existing evidence for antibody-pathogenicity (Table 3).

**Box 1:** Antibodies as biomarkers: current problems and future directions

Current problems	Possible solutions
**Antibodies as diagnostic biomarkers are not entirely specific**	
Rarely, low positive antibody titres can occur where the primary aetiology is not autoimmune. For example, GlyR antibodies were detected in Creutzfeldt-Jakob disease or genetic dystonia (Angus-Leppan *et al.*, 2013; Carvajal-Gonzalez *et al.*, 2014); NMDAR antibodies in serum or CSF of patients with Creutzfeld-Jakob disease (Fujita *et al.*, 2012; Mackay *et al.*, 2012); or MELAS syndrome (Finke *et al.*, 2012); and GABA_A_R-antibodies in genetically proven Huntington’s disease (Pettingill *et al.*, 2015). Similarly, neuronal antibodies without any clinical correlate have been found in healthy controls (Meinck *et al.*, 2001; Dahm *et al.*, 2014).	These findings highlight that antibody test results need to be interpreted with caution and clinical judgement.
	Methodological issues of antibody testing might be overcome by standardized tests and by international multicentre trials to establish the assays with the highest sensitivity and specificity.
	Diagnostic specificity can be increased e.g. by taking antibody titres into consideration, and by testing serum and CSF, and calculating intrathecal synthesis (particularly for GAD antibodies).
	It remains to be investigated if these antibodies could exert pathogenic effects in addition to the primary pathology.
**The controversial role of IgA and IgM antibodies**	
Pathogenic relevance was hitherto assigned only to antibodies of IgG subclass. NMDAR-antibodies of IgA-subtype were detected in patients with slow cognitive impairment in absence of inflammatory signs in MRI or CSF. Some patients responded to immunotherapy (Pruss *et al.*, 2012*b*).	There is emerging evidence of downregulation of NMDA receptors also by IgA and IgM antibodies in neuronal cell cultures (Pruss *et al.*, 2012*a*, *b*). However, their role remains controversial (Lancaster *et al.*, 2015) and further studies are warranted to determine if such patients should receive immunomodifying treatment.
**A need for predictive biomarkers to better guide therapeutic decisions**	
Antibody titres and clinical course correlate only in some (typically neuronal surface) antibodies. The correlation with serum antibody titres will possibly be poorer in diseases with predominant intrathecal synthesis (e.g. NMDAR, DPPX antibodies) than in disorders where the antibody is mainly generated in the serum (e.g. LGI1 antibodies).	In NMDAR-antibody encephalitis, the B-cell-attracting chemokine CXCL13 correlated with treatment responses and relapses (Leypoldt *et al.*, 2015).
	Another avenue to explore is FDG-PET imaging, which can show abnormalities even when the MRI is normal, and which often correspond to the clinical course (Kunze *et al.*, 2014).
**Commonly used treatment approaches are mainly empirical or based on expert opinions**	
There is a lack of evidence-based guidelines, mainly due to the relative rarity of these diseases.	Joint forces like international multicentre studies and registries with closely characterized patients would be desirable to investigate systematically the best treatment rationale.

MELAS = mitochondrial encephalomyopathy, lactic acidosis, and stroke-like episodes.

**Table 3:** Parallels between autoimmune and genetic conditions: common proteins either affected by gene mutations or targeted by autoantibodies, associated phenotypic spectrum and evidence for antibody pathogenicity

Protein	Function	Location	Genetic manifestation	Antibody manifestation	Evidence for antibody pathogenicity
(encoding gene)					
**Mixed movement disorders**					
NMDA receptor subunit GluN1	Critical subunit of NMDARs, key role in the plasticity of synapses	Ubiquitously on surface of neurons in the CNS	Early onset epileptic encephalopathy with dyskinesias, stereotypies, chorea, dystonia, myoclonus	NMDAR antibody encephalitis with epilepsy, dyskinesias, stereotypies, chorea, dystonia, myoclonus	Antibody titres correlate with clinical course; *in vitro* and *in vivo* evidence of antibodies causing receptor internalization (see text).
(*GRIN1*)					
Folate receptor	Folate uptake and supply	Expressed in several cell lines on the cell membrane; in the brain highly expressed in the choroid plexus	Early onset progressive movement disturbance, psychomotor decline, and epilepsy	Ataxia, myoclonus, epilepsy, psychomotor retardation, autism; children only	Antibodies block binding and uptake of folic acid (Ramaekers *et al.*, 2005).
(*FOLR1*)					
**SPSD**					
Glycine receptor alpha 1 subunit	Mediates postsynaptic inhibition	Surface of neurons in brainstem, spinal cord	Hereditary hyperekplexia	SPSD	Titres correlate with disease course; GlyR antibodies can activate complement and cause receptor internalization via lysosomal pathways *in vitro* (Carvajal-Gonzalez *et al.*, 2014).
(GlyRα1, *GLRA*)					
Glycine transporter 2 (GlyT2; *SLC6A5*)	Maintains a high presynaptic pool of glycine	Surface of neurons in brainstem, spinal cord	Hereditary hyperekplexia	SPSD	n.d.
**Cerebellar ataxia**					
Alpha-3 catalytic subunit of the Na+/K(+)-ATPase	Catalyses ATP-driven exchange of intracellular Na^+^ for extracellular K^+^ across the plasma membrane	Neuronal surface, various brain regions, including the basal ganglia, hippocampus, and cerebellum	RODP; AHC; rapid onset cerebellar ataxia; CAPOS	Cerebellar ataxia, gaze palsy, tetraparesis, gaze palsy, deterioration of visual acuity	Antibodies target a transmembrane domain which queries a direct pathogenic relevance; no experimental data (Scharf *et al.*, 2015).
(*ATP1A3*)					
Potassium channel, voltage-gated	CASPR2 associates with K_v_1.1 and is important for its juxtaparanodal clustering. K_v_1.1 mediates transmembrane potassium transport and contributes to the regulation of the membrane potential and nerve signalling	Highly expressed in cortex and cerebellum, and at Ranvier’s nodes in peripheral nerves	EA1 and myokymia	Peripheral nerve hyperexcitability, cerebellar ataxia, Morvan’s fibrillary chorea, limbic encephalitis	*In vitro* experiments with sera from patients with peripheral nerve hyperexcitability suggest that cross-linking of the channels by antibodies is likely to reduce K^+^ currents (Tomimitsu *et al.*, 2004).
(*VGKC*, K_v_1.1, *KCNA1*) and *CASPR2*					
Calcium channel, voltage-dependent, P/Q type	Mediates calcium influx, controls neuronal survival, excitability, plasticity and genetic expression, and mediate fast neurotransmitter release at synapses and neuromuscular junctions	Surface of Purkinje cells	SCA6; EA2; familial hemiplegic migraine	Cerebellar ataxia, Lambert-Eaton syndrome	Response to immunotherapy is poor, in line with patient IgG causing Purkinje cell death *in vitro* (McKasson *et al.,* 2016). Intrathecal injection of patient IgG induces cerebellar ataxia in mice. Antibodies of patients with ataxia target possibly other epitopes those in Lambert-Eaton myasthenic syndrome (Martin-Garcia *et al.,* 2013). VGCC antibodies are determined with a method liable to detect also intracellular antigens; it may be that the exact target(s) are still to be identified.
(VGCC P/Q type, *CACNA1*)					
Metabotropic glutamate receptor type 1	Important role in cerebellar development and synaptic plasticity, coupled to inositol phospholipid metabolism	Neuronal surface, highest expression in cerebellum, followed by cerebral cortex, thalamus, subthalamic nucleus, and amygdala	SCA13	Cerebellar ataxia	Correlation of antibody titres with symptoms (Sillevis Smitt *et al.*, 2000). Patient IgG block mGluR1 *in vitro* and cause ataxia in transfer experiments with rodents (Sillevis Smitt *et al.*, 2000). Brain pathology shows no CD8+ T cells (Coesmans *et al.*, 2003).
(mGluR1; *GRM1*)					
Glutamate receptor delta 2;	Associates with PKCγ and mGluR1; generates the appropriate time course of synaptic mGluR1 signalling; relevant for synaptogenesis in developing cerebellum; AMPA receptor trafficking	Surface of Purkinje cells	SCA18	Cerebellar ataxia	n.d.
(GluRδ2, *GRID2*)					
Protein kinase C gamma	Modulation of synaptic long-term potentiation and long-term depression, desensitizes mGlur1 by phosphorylation, induces AMPA receptor internalization	Intracellular cytosolic, Purkinje cells, cerebral cortex, hippocampus	SCA14	Cerebellar ataxia	n.d.
(PKCγ, *PRKCG*)					
Inositol 1,4,5-triphosphate receptor type 1 (*ITPR1*)	Intracellular IP_3_-gated channel that mediates calcium release from the endoplasmic reticulum	Intracellular, (mainly endoplasmic reticulum, cell body to dendritic spines); cerebellum, particularly Purkinje cells, hippocampus, caudate, putamen, and cerebral cortex	SCA15; SCA29	Cerebellar ataxia	n.d.

Carbonic anhydrase VIII (*CARP*, *CA8*)	ITPR1-binding protein that reduces the affinity of ITPR1 for IP3	Cytoplasma of cerebellar Purkinje cells	Cerebellar ataxia and mental retardation with or without quadrupedal locomotion 3	Cerebellar ataxia	n.d.

Glial fibrillary acidic protein (*GFAP*)	Intermediate filament proteins, with cyto-architectural functions and relevance for cell–cell communication	Cytoplasma of astrocytes	Alexander disease (leukodystrophy with psychomotor retardation, epilepsy, ataxia and spasticity)	Autoimmune GFAP astrocytopathy (meningoencephalomyelitis with encephalopathy, epilepsy, psychiatric symptoms, cerebellar ataxia, myelopathy)	n.d.

**Paroxysmal dyskinesias**					
Leucine-rich glioma-inactivated 1 (*LGI1*)	Forms with ADAM22 and ADAM23 a trans-synaptic complex, which interacts with K_v_1 potassium channels, PSD95 and AMPA receptors; role in regulating postnatal glutamatergic synapse development, AMPA receptor currents and density of axonal K_v_1 channels	Secreted from neurons, mainly in the hippocampus and neocortex	Familial temporal lobe epilepsy	Faciobrachial dystonic seizures, other forms of epilepsy, limbic encephalitis; chorea, parkinsonism	LGI1 antibodies interfere *in vitro* and *in vivo* with LGI1 binding to ADAM22 and ADAM23, causing a reversible reduction of synaptic AMPA receptors resulting in neuronal hyperexcitability (Ohkawa *et al.*, 2013)

ADAM = a disintegrin and metalloproteinase domain-containing protein; AHC = alternating hemiplegia of childhood; AMPA = α-amino-3-hydroxy-5-methyl-4-isoxazolepropionic; CAPOS = cerebellar ataxia, areflexia, pes cavus, optic atrophy, and sensorineural hearing loss; EA1/2 = episodic ataxia type 1/2; NMDAR = *N*-methyl-d-aspartic acid receptor; PSD95 = post-synaptic density protein 95; RODP = rapid onset dystonia parkinsonism; SCA = spinocerebellar ataxia.

### Neuronal surface antibodies

Antibodies against neuronal surface proteins might exert various effects upon binding, including complement activation and inflammatory cytotoxicity, antigenic modulation leading to receptor loss by internalization, or receptor blockade (Jain and Balice-Gordon, 2016). NMDAR antibodies are neuronal surface antibodies with *in vitro* and *in vivo* data supporting pathogenicity. NMDAR is an ionotropic glutamate receptor widely expressed in the brain and pivotal for long-term synaptic plasticity (Standaert *et al.*, 1994). *In vitro* and *in vivo* experiments have shown that NMDAR antibodies target the NR1 subunit of the receptor, causing receptor internalization by cross-linking and thereby a reduction of surface NMDAR density (Moscato *et al.*, 2014; Planaguma *et al.*, 2015). Upon removal of the antibodies, the receptor internalization is reversible, and residual deficits may be the result of glutamate excitotoxicity (Manto *et al.*, 2010). The distinct movement disorder associated with NMDAR antibodies, chorea and dyskinesias persisting in states of reduced consciousness, is also seen with ‘dissociative’ anaesthetics. Interestingly, these are NMDAR antagonists like ketamine or phencyclidine (Stamelou *et al.*, 2012). Furthermore, a genetic phenocopy of NMDAR-antibody encephalitis with mixed hyperkinetic movement disorders (chorea, dystonia, stereotypies, dystonia, oculogyric crises), seizures, and sleep cycle dysregulation is seen with mutations of *GRIN1*, the gene encoding the NR1 subunit of the NMDAR (Lemke *et al.*, 2016).

SPSD have also a genetic analogue in hereditary hyperekplexia, with which they share the clinical hallmark features of stiffness, spasms and exaggerated startle. Indeed, this clinical parallel inspired the discovery of GlyR antibodies and antibodies against the glycine transporter 2 (encoded by *SLC6A5*) (Hutchinson *et al.*, 2008; Balint *et al.*, 2015). GlyR antibodies specifically target the α1 glycine receptor subunit expressed on brainstem and spinal cord neurons, and both activate complement and cause receptor internalization via lysosomal pathways *in vitro* (Carvajal-Gonzalez *et al.*, 2014). The latter effect would be compatible with the clinical signs of decreased glycinergic neurotransmission and a loss of brainstem and spinal inhibition.

In contrast, the presumed pathophysiological mechanisms of DPPX antibodies, which also associate with SPSD, relate to increased CNS hyperexcitability mediated by downregulation of DPPX and K_v_4.2 in neuronal membranes as shown *in vitro* (Piepgras *et al.*, 2015). The antigen is widely expressed in the CNS and on the myenteric plexus, which matches the typically multifocal, combined presentations and chronic diarrhoea as hallmark features in DPPX-antibody-related disease.

Existing evidence for the pathogenic relevance of other neuronal surface antibodies and comparison with their respective genetic counterparts is summarized in Table 3.

### Antibodies against intracellular synaptic antigens

GAD antibodies also target a protein of inhibitory synapses, but their role in disease pathophysiology is more controversial. The antigenic target, glutamic acid decarboxylase-65 (GAD65), is the cytoplasmic, rate-limiting enzyme in the synthesis of GABA, a major inhibitory neurotransmitter in the CNS. GAD antibodies are the most frequent antibody in SPSD and cerebellar ataxia, but they also associate with temporal lobe epilepsy, limbic encephalitis, and type 1 diabetes (Gresa-Arribas *et al.*, 2015). Although a difference between epitopes associated with type 1 diabetes and SPSD/cerebellar ataxia was suggested, epitope mapping did not consistently reveal relevant differences between the antibodies pertinent to such different neurological phenotypes (Manto *et al.*, 2011; Fouka *et al.*, 2015; Gresa-Arribas *et al.*, 2015). The GAD antibody titres usually do not correlate with the clinical course, and the response to immunotherapy is highly variable. These observations have questioned their pathogenicity. Some studies have identified co-occurring, potentially pathogenic neuronal surface antibodies in patients with GAD antibodies, but it is questionable if this is sufficient to explain the varied neurological manifestations (Chang *et al.*, 2013; Petit-Pedrol *et al.*, 2014; Gresa-Arribas *et al.*, 2015). Pathological findings from patients with SPSD or encephalitis with GAD antibodies substantiate a T-cell involvement, and suggest that GAD-antibody-related disease represents an intermediate between neuronal surface antibodies and those directed against cytoplasmic/nuclear antigens (Witherick *et al.*, 2011; Bien *et al.*, 2012). Whereas *in vitro* experiments yielded contradictory evidence regarding the possible internalization of GAD-antibodies (Hampe *et al.*, 2013; Gresa-Arribas *et al.*, 2015), transfer experiments in rodents were able reproduce some evidence of pathogenicity (Geis *et al.*, 2011).

Similar transfer experiments of purified IgG have shed a new light on amphiphysin antibodies. The antigen has a pivotal role for clathrin-mediated endocytosis, a mechanism to compensate for the fast exocytosis of neurotransmitters by recycling synaptic vesicles, which is particularly important in GABAergic interneurons. Amphiphysin-IgG reduced presynaptic GABAergic inhibition, leading to stiffness and spasms in rodents (Sommer *et al.*, 2005; Geis *et al.*, 2010). Neurons internalized the antibodies and reduced the presynaptic vesicle pool (Werner *et al.*, 2016). Further studies will have to elucidate if the transient presentation of the ‘surface-moonlighting’ synaptic antigens during endocytosis suffices to generate pathology (Irani, 2016).

### Antibodies against intracellular cytoplasmic/nuclear antigens

Autoantibodies against intracellular antigens are not considered pathogenic, as their target is inaccessible *in vivo*. Existing evidence suggests that autoreactive T cells are mediating the disease process, characterized by lymphocytic infiltration and damage of neuronal structures (Lancaster and Dalmau, 2012). This group of antibodies includes those against nuclear or nucleolar antigens, like Hu or Ma, which have a diffuse expression in the CNS and various associated syndromes.

**Figure 3:**
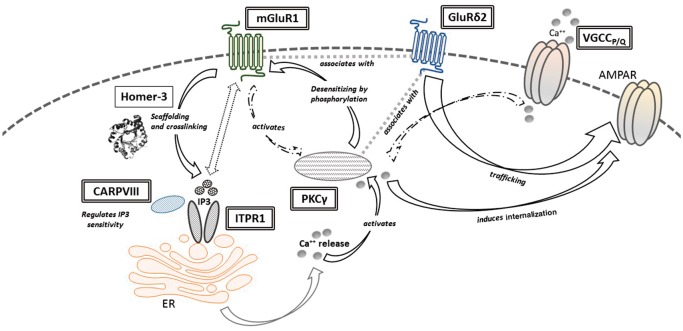
**Proteins of the calcium homeostasis and signalling network in Purkinje cells: parallels of genetic cerebellar ataxias and antibody-related autoimmunity**. Upon parallel fibre stimulation, glutamate is released and binds to mGlur1, a G-protein-coupled surface receptor highly expressed at the perisynaptic site of Purkinje cells and involved in mediation of slow excitatory potentials and long-term depression. Its intracellular domain in turn interacts with Homer-3, which is a scaffolding protein relevant for mGlur1 clustering, and which crosslinks mGluR1 with ITPR1 in the smooth endoplasmic reticulum. Upon glutamate binding, mGluR1 activates of phospholipase C, an enzyme that cleaves phosphatidylinositol 4,5-bisphosphate in the plasma membrane to produce diacylglycerol and inositol trisphosphate (IP3). IP3 binds to ITPR1 and thereby induces calcium release from the endoplasmatic reticulum, which in turn activates protein kinase C γ (PKCγ) that desensitizes mGluR1 by phosphorylation and induces internalization of AMPA receptors (AMPAR). GluRδ2 is a key binding partner for mGluR1 and PKCγ, relevant for synaptic mGluR1 signalling and also involved in AMPAR trafficking. Similarly, activation of climbing fibres opens voltage gated calcium channels (VGCC) which mediate calcium influx and contribute to the signalling cascade, which results in reduction of AMPAR sensitivity at the synapse. Proteins that are targets of antibodies associated with cerebellar ataxia are in single-lined boxes, proteins that are also affected by mutations in genetic ataxias (Table 3) are highlighted in double-lined boxes, and existing evidence with regards to their pathogenic role is discussed in Table 3. AMPAR = α-amino-3-hydroxy-5-methyl-4-isoxazolepropionic acid receptor; CARP VIII = carbonic anhydrase VIII; ER = endoplasmic reticulum; GluRd2 = glutamate receptor delta 2; PKCg = protein kinase C gamma.

A subgroup of these antibodies, however, target cytoplasmic antigens and specifically associate with cerebellar ataxia with Purkinje cell degeneration. Indeed, these conditions have genetic counterparts involving a functional network of calcium homeostasis and signalling in Purkinje cells, and therefore demonstrate molecular parallels between genetic conditions and autoantibodies considered as non-pathogenic (Table 3 and Fig. 3). Although IgG uptake by Purkinje cells has been reported (Hill *et al.*, 2009; Greenlee *et al.*, 2010), further experiments substantiating a possible pathophysiological role of these antibodies are lacking.

Similarly, antibodies against transglutaminase 6 (TG6), which is *inter alia* expressed in the cytoplasm of Purkinje cells, have a genetic counterpart in SCA35 (Wang *et al.*, 2010). TG6 antibodies have been described in patients with gluten sensitivity, but their sensitivity, specificity and diagnostic utility as well as their pathophysiological role remain very controversial (Hadjivassiliou *et al.*, 2008; Boscolo *et al.*, 2010; Lindfors *et al.*, 2011; Hadjivassiliou *et al.*, 2013; McKeon *et al.*, 2014; Stenberg *et al.*, 2014).

### The role of neuronal antibodies in neurodegeneration: player, bystander or biomarker?

The recent discovery of neuronal surface antibodies against IgLON5 in defining a novel tauopathy has more closely apposed the boundaries between neurodegeneration and neuroimmunology (Sabater *et al.*, 2014).

The IgLON5-antibody-linked tauopathy is characterized by prominent sleep movement disorders, first and foremost by a non-REM sleep parasomnia with simple or finalistic movements, resembling daytime activities such as eating, drinking or manipulating objects. Other sleep abnormalities included RBD, and periodic limb movements of sleep.

Breathing difficulties like sleep apnoea or stridor, leading to respiratory insufficiency and often severe enough to require a tracheostomy, appear to be another hallmark feature of this disease. Bulbar symptoms, namely dysarthria, dysphagia and vocal cord paresis, are common findings. Patients may be disabled by a progressive and disabling gait instability with postural reflex loss, which together with a vertical supranuclear gaze palsy give rise to a PSP-like presentation (Gaig *et al.*, 2017; Hoglinger *et al.*, 2017). The range of abnormal eye movements extends however to horizontal gaze paresis, saccadic intrusions, and nystagmus (Sabater *et al.*, 2014; Gelpi *et al.*, 2016). On the other hand, ocular or appendicular cerebellar signs, nocturnal stridor and dysautonomia including orthostatic hypotension, can resemble multiple system atrophy. Possible signs of dysautonomia comprise also urinary symptoms, episodic intense transpiration, cardiac arrhythmias and central hypoventilation. The phenotypic spectrum is indeed broad, and includes also a Huntington’s disease lookalike with chorea, myoclonus and cognitive decline.

Disease onset ranged between 48–77 years, and disease duration spanned from 2 months to 12 years. The causes of death were respiratory failure or sudden death during sleep or wakefulness. Notably, brain pathology showed an absence of inflammatory infiltrates but widespread accumulation of hyperphosphorylated three and four repeat tau aggregates in neurons, and neuronal loss predominantly in the hypothalamus and the brainstem tegmentum (Sabater *et al.*, 2014; Gelpi *et al.*, 2016). There was a cranio-caudal gradient of severity until the upper cervical cord. These findings suggest neurodegeneration as the primary disease mechanism, which would fit with the observed absence of a significant response to immunotherapy. However, all genotyped patients had HLA-DQB1*0501 and HLA-DRB1*1001 alleles, suggesting a genetic susceptibility for autoimmunity (Sabater *et al.*, 2014, 2016).

How can these seemingly contradictory and puzzling findings be reconciled? Little is known about the physiological function of IgLON5, a cell adhesion molecule on the neuronal surface. It belongs to the immunoglobulin superfamily and functions in neuronal path-finding and synaptic formation during brain development (Sanz *et al.*, 2015). The IgLON5 antibodies target the extracellular domain of the protein and are predominantly of the IgG4 subtype, which is assigned anti-inflammatory properties. To a lesser extent, patient sera contained co-existent IgG1 antibodies, which caused internalization of IgLON5 in neuronal cultures (Sabater *et al.*, 2016). This effect was not seen with IgG4-subtype antibodies. Thus, the role of the IgLON5 antibodies is still unclear. Perhaps antibody-mediated downregulation of IgLON5 could disrupt its interaction with the internal cytoskeletal network and induce tau accumulation and hyperphoshphorylation, leading to neurodegeneration, which at the time of manifestation may no longer be amenable to immunotherapy (Gelpi *et al.*, 2016). On the other hand, the tau accumulation may indirectly lead to neuronal autoimmunity in susceptible individuals, and have broader implications as a paradigm for the role of inflammation in neurodegeneration.

## Conclusions and future directives

The ever-expanding, partly overlapping, spectrum of antibodies in movement disorders, and knowledge about the clinical phenotypes is key to identify patients who may benefit from therapy. Current challenges (Box 1) relate to the use of antibodies as biomarkers. Presently, treatment approaches of immuno-suppression or -modulation are empirical or based on expert consensus, as there is a lack of randomized controlled trials and available evidence is limited to a few observational studies (Graus *et al.*, 2016). The underlying immunopathophysiology can inform treatment decisions and prognosis (Fig. 2). In disorders where neuronal surface antibodies are the main players, overall response to immunotherapy is usually good, albeit varied. For example, usually, patients with LGI1 antibodies show an exquisite response to corticosteroids, whereas approximately half of the patients with NMDAR antibodies respond insufficiently to ‘first line’ immunotherapy (corticosteroids, plasma exchange, intravenous immunoglobulins) (Irani *et al.*, 2013; Titulaer *et al.*, 2013), which may be partly related to extra- versus intrathecal antibody synthesis. Regarding intracellular synaptic antibodies, there has been one placebo-controlled cross-over trial showing beneficial effects of intravenous immunoglobulin in patients with stiff person syndrome and GAD antibodies (Dalakas *et al.*, 2001) but, overall, treatment response in GAD-antibody-related disease is mixed and unpredictable (Arino *et al.*, 2014; Martinez-Hernandez *et al.*, 2016).

**Figure 4:**
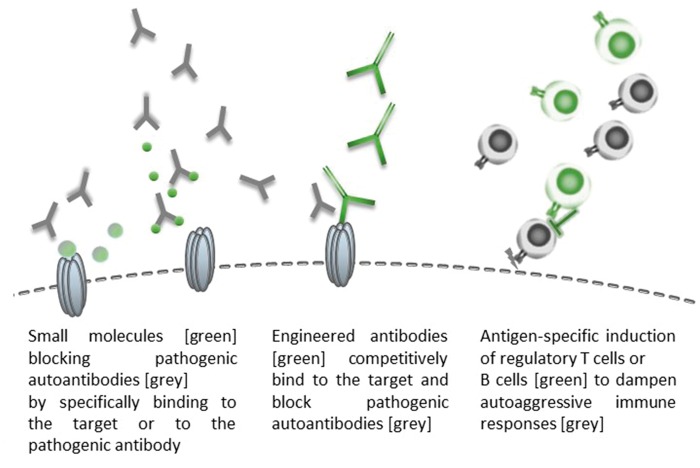
**Potential future treatment approaches with antigen-specific immunotherapy**.

The main drawback of the present immunotherapies is that they are potentially toxic: they widely suppress the immune system and lead to a higher risk of infections, possibly with fatal outcomes. A future, improved treatment option may be more tailored, antigen-specific immunotherapy (Fig. 4). In primarily antibody-mediated disease, targeted blocking of the patients’ autoantibodies with small molecules could attenuate the effect of the autoantibodies. Another possibility is engineering a non-pathogenic, monoclonal antibody that binds to the same target and competitively displaces the autoantibodies, thereby preventing cytotoxicity. Persuasive precedents exist in neuromyelitis optica caused by AQP4 autoantibodies, where the antiviral agent arbidol can serve as such a small blocking molecule, or ‘aquaporumab’ as a competitive and protective antibody (Verkman *et al.*, 2013).

In T-cell-mediated disease, this may involve expanding the antigen-specific regulatory networks, in particularly regulatory T cells (Clemente-Casares *et al.*, 2016). Autoimmune disease-relevant peptides can induce antigen-specific regulatory T cells and reverse autoimmune inflammation, as shown in mice humanized with lymphocytes from type 1 diabetes and treated with nanoparticles coated with human GAD65 (Clemente-Casares *et al.*, 2016).

A different approach is to target the pathophysiological cascades downstream from the antibody–antigen interaction. A molecular understanding, which has been developed through genetic diseases over the past decades, could now be used to think about new therapeutic approaches for antibody-related disease. Such a molecular, pathway-specific approach seems feasible in NMDAR-antibody encephalitis: NMDAR antibodies disrupt the interaction between NMDAR and ephrin-B2 receptor, which eventually leads to displacement of NMDAR to extrasynaptic sites before they are internalized. Ephrin-B2 is the ligand of the ephrin-B2 receptor, which stabilizes and retains NMDAR at the synapse. Administration of ephrin-B2 counteracts this loss of extrasynaptic and synaptic NMDAR both *in vitro* and *in vivo* in the NMDAR-antibody transfer mouse model (Mikasova *et al.*, 2012; Planagumà *et al.*, 2016). Notably, aberrant NMDAR trafficking is also pivotal in the pathophysiology of Huntington’s disease (Daggett and Yang, 2013), and reagents that regulate human ephrin-like receptors are under investigation for treatment of Huntington’s disease (Ramakrishnan, 2003). Clinical practice offers another paradigm of a similar treatment approach in genetic and autoimmune neurology where 4-aminopyridine has proved beneficial in two disorders affecting P/Q type voltage-dependent calcium channels: episodic ataxia type 2 due to P/Q calcium channel gene mutations and Lambert-Eaton syndrome due to P/Q calcium channel autoantibodies (Strupp *et al.*, 2011). There are a number of antibodies associated with cerebellar ataxia that target proteins, which are functionally relevant parts of a calcium signalling network in Purkinje cells, and also affected by mutations causing genetic ataxias (Table 3 and Fig. 3). Thus, an interdisciplinary approach with focus on similar pathophysiological pathways in immunological, genetic or degenerative disorders will cross-fertilize our endeavours to better understand and treat neurological diseases.

## Funding

B.B. is supported by the EAN research fellowship programme. S.R.I. is supported by a Wellcome Trust Intermediate Clinical fellowship, the British Medical Association – Vera Down Research Grant, the UCB-Oxford Alliance, and is part of the Oxford Epilepsy Research Group. SRI is a coapplicant and receives royalties on patent application WO/2010/046716 entitled ‘Neurological Autoimmune Disorders’. The patent has been licensed to Euroimmun AG for the development of assays for LGI1 and other VGKC-complex antibodies. A.V. received grant support from GlaxoSmithKline, and royalties from patents related to MuSK antibodies for diagnosis of myasthenia gravis (U.K. patent no., PCT/GB01/02661; licensed to Athena Diagnostics) and LGI1 and CASPR2 antibodies for autoimmune encephalitis (U.K. patent no., PCT/GB2009/051441; licensed to Euroimmun). K.P.B. holds research grants from NIHR RfPB, MRC Wellcome Strategic grant (Ref. no.: WT089698) and PD UK (Ref. no.: G-1009), and has received honoraria/financial support to speak/attend meetings from GSK, Boehringer-Ingelheim, Ipsen, Merz, Sun Pharma, Allergan, Teva Lundbeck and Orion pharmaceutical companies. K.B. receives royalties from Oxford University press and a stipend for MDCP editorship. H.M.M. reports no disclosures.

## Supplementary material

Supplementary material is available at *Brain* online.

## Supplementary Material

Supplementary Video 1

## Abbreviations

OMS: opsoclonus-myoclonus syndrome
PANDAS: paediatric autoimmune neuropsychiatric disorders associated with streptococcal infections
RBD: REM sleep behaviour disorder
SPSD: stiff person spectrum disorders
